# Editorial

**DOI:** 10.17886/RKI-GBE-2017-086

**Published:** 2017-08-30

**Authors:** Susanne Jordan, Thomas Ziese, Ursula von Rueden

**Affiliations:** 1 Robert Koch Institute, Department of Epidemiology and Health Monitoring, Berlin; 2 Federal Centre for Health Education, Unit 2-25: Research, Quality Assurance, Cologne, Germany

## Abstract

In November 2016, during a workshop organised by the Robert Koch Institute together with the Federal Centre for Health Education, we presented and discussed activities and models surrounding prevention reporting with health reporting representatives from the federal states. The motive for the event was the prevention report, which the National Prevention Conference will prepare every four years beginning in 2019 in order to document, monitor and evaluate its activities. The workshop revealed the desire of stakeholders to discuss survey methods and indicators and harmonise the different reporting systems in the long-term.

Following the adoption of the Preventive Health Care Act in 2015, the Robert Koch Institute and the Federal Centre for Health Education organised a workshop on prevention reporting with health reporting representatives from the federal states. The social insurance carriers, the forum gesundheitsziele.de and researchers with scientific expertise in the field were also invited. Overall, around fifty experts convened on 24-25 November 2016 in Berlin to discuss their experiences and the challenges prevention reporting faces to meet the requirements laid out by the Preventive Health Care Act. The introductory presentations focused on questions of methodology for prevention reporting and presented approaches and options to implement these approaches.

The workshop was organised against the backdrop of the legal demands arising from the Preventive Health Care Act for the institutions organised in the National Prevention Conference to present an initial report on prevention across all relevant institutions by 2019. The RKI is to provide the monitoring data, and the federal states can share regional results from their health reporting. The report aims to provide a comprehensive assessment of settings- and workplace-related preventive and health promotion measures in Germany, based on which, following the second report (in 2023), developments over time can be shown. Social insurance carriers will provide quantitative data on expenditure, services provided, forms of access and the target groups/people reached for the targets Grow up healthy, Living and working healthy and Healthy ageing. Moreover, they will provide information on quality assurance procedures and on collaboration efforts in the implementation of services (see Liedtke et al. 2017).

Regarding questions of the methodology to be applied by national prevention reporting, different approaches were presented, which, in line with the Public Health Action Cycle approach, should allow us to combine data-based prevention needs, prevention strategies with their health targets and evaluation/impact assessment. This aims to counter the low degree to which research results are currently being translated into evidence-based preventive practices. For prevention reporting, using impact models and developing suitable indicators for health promotion and disease prevention thereby remain important prerequisites (see von Rueden et al. 2017, Jordan et al. 2017). Regarding the first report by the National Prevention Conference, the demand was voiced to report especially on strategies and impacts of measures to create health-promoting settings, and that a report would have to be produced by an independent commission (see Geene 2017). Various workshop contributions emphasised that prevention reporting should include data on interventions, policy and media monitoring in addition to health monitoring data. National-level prevention reporting should build on the expertise provided by platforms and institutions such as gesundheitsziele.de to evaluate individual targets (see Maschewsky-Schneider 2017). State-level health expenditure accounts could provide information on the sums that individual states have spent on disease prevention and health protection (see Meise et al. 2017).

Initial steps and various approaches towards comprehensive prevention reporting are underway in the federal states. Based on their Health Map (Gesundheitsatlas), Baden-Württemberg has developed a health barometer. This assessment system allows a comparison between individual districts based on the score or ranking they achieve for specific indicators (see Würz 2017). In 2013, Berlin’s state health conference initiated intervention reporting. This consists of surveying data to describe the implemented measures for health promotion and disease prevention. Based on results from an initial survey in kindergartens in 2013, possible applications for intervention reporting were presented. The initial experiences show that intervention reporting can support the planning of measures, particularly with regard to aspects that compensate for social differences, and can provide information on the type and extent, though not on the efficacy, of these measures (see Bettge et al. 2017). Bavaria is developing prevention reporting that will include the results from an online survey on prevention measures that was conducted in 2014/2015 among 600 stakeholders. This presentation, too, pointed out that drawing conclusions about the impact of health promotion and primary prevention measures on the health of the population was not possible (see Reisig et al. 2017). The presentation from Hamburg (see Saier 2017) also emphasized the difficulty of assessing the effectiveness of interventions, as effects can only be measured in the long-term and evaluation reports are not standardised and difficult to compare. Prevention reporting therefore needs to determine the conditions, structures and processes that lead to successful interventions (best-practice models) and develop indicators capable of measuring the quality of processes and results. Analogous to the national prevention report, the city of Hamburg will also provide its own report every four years. North Rhine-Westphalia is considering initially using indicators already available from district-level health reporting and municipal health reports to assess demand. As the services mandated by the Preventive Health Care Act will need to be delivered in the context of needs identified at the settings level, this provides a link to municipal health reporting. An example from Saxony-Anhalt illustrates the role played by the public health service (ÖGD). ÖGD screenings in kindergartens and schools should become part of prevention reporting (see Wahl 2017).

Through the Preventive Health Care Act, the scientific and health policy discussion on meaningful prevention reporting has significantly gained momentum. The law provides an incentive to advance and develop questions of methodology. We need to discuss suitable survey methods and indicators to measure the implementation, scope and impact of measures for prevention and health promotion. Equally, there is a certain amount of pressure to establish prevention reporting at different levels. Federal, state and local authorities require this data to further develop prevention strategies. This is linked to the opportunity and challenge for reporting systems at the federal, state and local level to strengthen their collaboration and coordination. This workshop clearly showed that the concept for the first prevention report of the National Prevention Conference so far has only few points of reference with the reporting systems already in place in some federal states and differs from various scientific impact models of prevention and health promotion. In the long-term, it would be desirable to make use of synergies and harmonise the different systems.

